# Value of Information: Sensitivity Analysis and Research Design in Bayesian Evidence Synthesis

**DOI:** 10.1080/01621459.2018.1562932

**Published:** 2019-04-30

**Authors:** Christopher Jackson, Anne Presanis, Stefano Conti, Daniela De Angelis

**Affiliations:** a MRC Biostatistics Unit, University of Cambridge, UK;; b NHS England, Leeds, UK

**Keywords:** Decision theory, Research prioritization, Uncertainty

## Abstract

Suppose we have a Bayesian model that combines evidence from several different sources. We want to know which model parameters most affect the estimate or decision from the model, or which of the parameter uncertainties drive the decision uncertainty. Furthermore, we want to prioritize what further data should be collected. These questions can be addressed by Value of Information (VoI) analysis, in which we estimate expected reductions in loss from learning specific parameters or collecting data of a given design. We describe the theory and practice of VoI for Bayesian evidence synthesis, using and extending ideas from health economics, computer modeling and Bayesian design. The methods are general to a range of decision problems including point estimation and choices between discrete actions. We apply them to a model for estimating prevalence of HIV infection, combining indirect information from surveys, registers, and expert beliefs. This analysis shows which parameters contribute most of the uncertainty about each prevalence estimate, and the expected improvements in precision from specific amounts of additional data. These benefits can be traded with the costs of sampling to determine an optimal sample size. Supplementary materials for this article, including a standardized description of the materials available for reproducing the work, are available as an online supplement.

## 1 Introduction

Bayesian modeling is a natural paradigm for decision making, in the presence of uncertainty, based on multiple sources of evidence. However, as more data sources, parameters, and assumptions are built into a model, it becomes harder to see the influence of each input or assumption. The modeling process should involve an investigation of where the weak parts of the model are, to identify which uncertainties in the model inputs contribute most to the uncertainty in the final result or decision (*sensitivity analysis*). We might then want to assess and compare the potential value of obtaining datasets of specific designs or sizes to strengthen different parts of the model. Furthermore, we may want to formally trade-off the costs of sampling with the resulting expected improvement to decision making.

Annual estimation of HIV prevalence in the United Kingdom has, for several years, been based on a Bayesian synthesis of evidence from various surveillance systems and other surveys (Goubar et al. 2008; Presanis et al. 2010; De Angelis et al. 2014; Kirwan et al. 2016). This is an example of a class of problems called *multiparameter evidence synthesis* (MPES) (e.g., Ades and Sutton 2006), where the quantities of interest cannot be estimated directly, but can be inferred from multiple indirect data sources linked through a network of model assumptions that can be expressed as a directed acyclic graph. Markov chain Monte Carlo (MCMC) is typically required to estimate the posterior. The HIV MPES model is used to inform health policies, thus it is crucial to be able to assess sensitivity to uncertain inputs and to indicate how the model could be strengthened with further data.

These dual aims can be achieved with *value of information* (VoI) analysis, a decision-theoretic framework based on expected reductions in loss from future information. The concepts of VoI were first set out in detail by Raiffa and Schlaifer (1961), while Parmigiani and Inoue (2009) give a more recent overview. The expected value of *partial perfect information* (EVPPI) is the expected reduction in loss if the exact value of a particular parameter or parameters $\theta_{0}$ were learnt, also interpreted as the amount of decision uncertainty that is due to $\theta_{0}$. The expected value of *sample information* (EVSI) is the expected reduction in loss from a study of a specific design. The EVSI can be traded off with the costs of data collection to give the *expected net benefit of sampling (ENBS)*. Therefore, as well as recommending a policy based on minimising expected loss under the *current* model and data, the decision-maker may also recommend collecting *further* data according to a design which minimises the ENBS.

These concepts have been applied in various forms in three distinct areas: health economics, computer modeling and Bayesian design. In health economic modeling, there is a large literature on calculation and application of VoI, see, for example, Felli and Hazen (1998); Willan and Pinto (2005); Claxton and Sculpher (2006); Welton et al. (2008). The model output is then the expected net benefit of each alternative policy, a known deterministic function g(θ) of uncertain inputs θ, and the decision problem is the choice of policy that minimises g(θ). In computer modeling, see, for example, Oakley and O’Hagan (2004) and Saltelli et al. (2004), the influence of a particular element $\theta_{0}$ of θ is calculated as the expected reduction in var(g(θ)), if we were to learn $\theta_{0}$ exactly. This is equivalent to the EVPPI for $\theta_{0}$ under a decision problem defined as point estimation of g(θ) with quadratic loss (Oakley and O’Hagan 2004). The decision-theoretic view of Bayesian experimental design also has a long history, see, e.g. Lindley (1956); Bernardo and Smith (1994); Chaloner and Verdinelli (1995); Berger (2013), and a recent review of the computational challenges by Ryan et al. (2016).

However, the current tools in any one of these three areas cannot be applied directly to MPES. First of all, it is not always feasible or desirable to make a discrete decision with a quantifiable loss, as in health economic modeling. Instead, the aim of evidence synthesis is often to estimate one or more quantities. For a scalar quantity of interest, we might then define the “loss” as the posterior variance of this quantity, as Oakley and O’Hagan (2004) described in the computer modeling context. In computer modeling, however, tools to estimate the expected value of a proposed study to learn a particular $\theta_{0}$ more precisely have not been developed, and it is not clear what an appropriate loss for a vector of model outputs would be. Challenges also arise with computation. Current methods for computing the expected variance reduction in the computer modeling field (Sobol’ 2001; Saltelli et al. 2004) assume the output is an explicit function g(θ) of the inputs, therefore do not apply in MPES, where this function is unknown and the outputs must be estimated by MCMC. For Bayesian design, Ryan et al. (2016) reviewed methods where evaluating the expected utility of a design (equivalent to the EVSI) is relatively inexpensive, so that maximizing the utility over a complex design space is feasible. However, this can again be difficult with MCMC. Given a sample from the posterior p(θ|x), potential future datasets y under a specific design can be simulated cheaply from the posterior predictive distribution, but then to obtain the expected utility, the posterior p(θ|x,y) needs to be repeatedly updated for different y, which is feasible with Monte Carlo only for smaller problems (e.g., Han and Chaloner 2004).

Here, we use and extend methods from health economics, computer modeling, and Bayesian design to devise a new VoI framework for sensitivity analysis and research design in evidence syntheses based on graphical models fitted by MCMC. This is a broader class of models than those typically used in health economics or computer modeling, since the model “output” is not necessarily a known function of the inputs, but depends on the model parameters θ and observed data x through a network of statistical models or deterministic functions, potentially with hierarchical relationships. We apply this new VoI framework to the part of the HIV prevalence estimation model that estimates prevalence in men who have sex with men (MSM), in London. Here, the decision problem is point estimation of a single scalar or a vector of parameters, followed by the choice of what extra data should be collected in the future. We use ideas from Bayesian design to choose appropriate loss functions in this context. We also generalize methods of computing EVPPI (Strong, Oakley, and Brennan 2014) and EVSI (Strong et al. 2015), developed for finite choices in health economics, to a broader class of decision problems, including point estimation. The method for computing EVSI enables the expected utility over all potential y to be estimated cheaply without an additional level of simulation, assuming only that the information provided by y can be represented as a low-dimensional sufficient statistic T(y).

In Section 2, we describe the general MPES model, and define the expected VoI under different decision problems and loss functions, and in Section 3, we present methods to compute them. In Section 4, we describe the model for HIV prevalence estimation, and in Section 5, we use VoI to identify the areas of greatest uncertainty in this model and determine what specific data should be collected to improve the precision of the estimates of various subgroup-specific prevalences. Finally, we discuss potential extensions to the methods and application and the associated challenges.

## 2 Theory and Methods

### 2.1 Bayesian Graphical Modeling for Evidence Synthesis

**Fig. 1:**
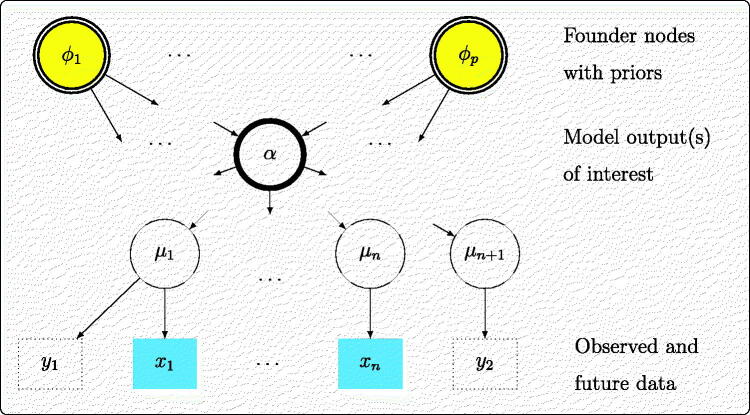
Directed acyclic graph for Bayesian evidence synthesis.

In our motivating applications, the general model can be represented as a directed acyclic graph (Figure 1) in the standard way, see, for example, Lauritzen (1996). Nodes in the graph may represent scalar or vector quantities. A set of datasets $x=\{x_{1},\ldots ,x_{n}\}$ is observed, most generally from *n* different sources. These data are assumed to arise from statistical models with parameters $\mu_{1},\ldots ,\mu_{n}$ respectively, collectively denoted μ. The “founder nodes” of the graph are denoted $\phi =(\phi_{1},\ldots ,\phi_{p})$ and given a joint prior distribution ϕ∼p(.) which may also include substantive information. The full set of unknowns is denoted θ. Most simply, the μ could equal the ϕ or be related to the ϕ through deterministic functions, so that θ=ϕ. More generally, some of the relationships in the graph could be stochastic, defining a hierarchical model, where the μ themselves arise from a distribution with parameters given by the ϕ or descendants of ϕ. The vector of unknowns θ would then comprise ϕ and the stochastic descendants of ϕ such as random effects.

We further denote α as an intermediate node in the graph, the model “output,” which is used for decision making. This could be any unknown quantity, including one of the μ or ϕ, a function of these, or a prediction of new data. We may also plan to collect additional data, either from the same source as one of the existing datasets (e.g., *y*_1_ in Figure 1), or from a new source informing a parameter $\mu_{n+1}$ on which no direct data (*y*_2_) were available.

**Fig. 2:**
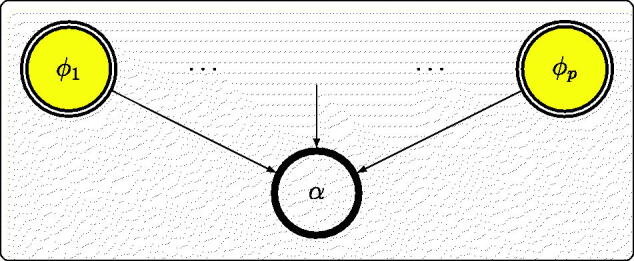
Graph representing a known deterministic model.

This DAG (Figure 1) is a generalization of the typical structure (Figure 2) used in computer modeling (Oakley and O’Hagan 2004) where the output α is a known (usually complicated) deterministic function of uncertain model inputs ϕ, which are given substantive priors that may be derived separately from data.

### 2.2 Expected VoI: Definitions

In a general decision-theoretic framework, the purpose of the model is to choose a decision or action *d* from a space of possible decisions D, to minimise an expected loss $E_{\theta }(L(d,\theta ))$, with the expectation taken with respect to the posterior distribution of θ. Let α=α(θ) be the minimal subset or function of θ necessary to make the decision, so that $E_{\theta }(L(d,\theta ))=E_{\alpha }(L(d,\theta ))$, ∀d∈D. For example, the purpose could be the choice of decision *d* among a finite set D={1,…,D} expected to minimize a loss defined as a function of the parameters, so that α would be a vector with *D* components $\alpha_{d}=f_{d}(\theta )=L(d,\theta )$. This is the typical situation in health policy decisions (e.g., Claxton and Sculpher 2006), where a treatment *d* is chosen to maximize a measure of utility such as expected quality-adjusted survival. Alternatively, as in our examples, the decision could simply be the choice of a point estimate $\hat{\alpha }$ of some parameter α, in which case the decision space D is the support of α (see Section 2.3). Alongside making a decision, we wish to also determine where further research should be prioritized to reduce the uncertainty about the decision, and given the costs of data collection, to determine the optimal design of further research (see Section 2.4).

For general decision problems, let $d^{*}=\text{arg}\;{\text{min}}_{d}E_{\theta }(L(d,\theta ))$ be the optimal decision under *current* knowledge about θ, represented by the posterior distribution p(θ|x). Suppose now we are in a position to collect new information. Let $d_{y}^{*}$ be the optimal decision given further knowledge of a quantity y (either parameters or potential data) that informs α, so that the updated posterior would be p(θ|x,y). We define the following quantities.

1. The *expected value of perfect information* (EVPI) is the expected loss of the decision $d^{*}$ under current information, minus the expected loss for the decision $d_{\alpha }^{*}$ we would make if we knew the true α (Raiffa and Schlaifer 1961).
  $$\text{EVPI}=E_{\theta }(L(d^{*},\theta ))-E_{\theta }(L(d_{\alpha }^{*},\theta ))$$
  Since additional information is always expected to reduce the expected loss of the optimal decision (Parmigiani and Inoue 2009), the EVPI is an upper bound on the expected gains from any new information.
2. The *expected value of partial perfect information* (EVPPI) for a particular parameter ϕ is the expected reduction in loss if ϕ were to be learnt precisely. Since this precise value is not yet known, an expectation must be taken over all possible values.
  (1) $$\text{EVPPI}(\phi )=E_{\theta }(L(d^{*},\theta ))-E_{\phi }[E_{\theta |\phi }(L(d_{\phi }^{*},\theta ))],$$
  where $d_{\phi }^{*}$ is the optimal decision if ϕ were known. This is an upper bound on the potential value of data y which inform only ϕ. In a graphical model, this means data y that are conditionally independent of θ given ϕ, for example $y=y_{1}$ and $\phi =\mu_{1}$ in Figure 1.
3. The *expected value of sample information* EVSI(y) is the reduction in loss we would expect from collecting an additional dataset y of a specific design
  (2) $$\text{EVSI}(y)=E_{\theta }(L(d^{*},\theta ))-E_{y}\left[E_{\theta |y}(L(d_{y}^{*},\theta )\right].$$

The inner expectation is now with respect to the updated posterior distribution of θ|y, after learning y as well as the existing data x, or “preposterior” (Berger 2013).

### 2.3 VoI in Different Decision Problems

#### Finite-Action Decisions

For a choice of *d* among a finite set {1,…,D} with loss $L(d,\theta )=\alpha_{d}$ and $\alpha =\{\alpha_{1},\ldots ,\alpha_{d},\ldots ,\alpha_{D}\}$, the expected loss with current information is $\min_{d}\{E_{\alpha }(\alpha_{d})\}$, so (Raiffa and Schlaifer 1961)

(3) $$\begin{array}{c} \text{EVPI}=\underset{{}_{d}}{\min}\{E_{\alpha }(\alpha_{d})\}-E_{\alpha }\underset{{}_{d}}{\min}\{\alpha_{d}\},\\ \text{EVPPI}(\phi )=\underset{{}_{d}}{\min}\{E_{\alpha }(\alpha_{d})\}-E_{\phi }\underset{{}_{d}}{\min}\{E_{\theta |\phi }(\alpha_{d})\},\\ \text{EVSI}(y)=\underset{{}_{d}}{\min}\{E_{\alpha }(\alpha_{d})\}-E_{y}\underset{{}_{d}}{\min}\{E_{\alpha |y}(\alpha_{d})\}, \end{array}$$

##### Point estimation

When the decision is the choice of a point estimate $\hat{\alpha }$ of a vector of parameters α, with quadratic loss

(4) $$L(\hat{\alpha },\alpha )={\left(\hat{\alpha }-\alpha \right)}^{T}H(\hat{\alpha }-\alpha )$$

for a symmetric, positive-definite *H*, the optimal estimate with current information is the posterior mean, $\hat{\alpha }=E_{\alpha }(\alpha )$.

For a scalar α=α and *H* = 1, the expected loss is var(α) under current information and zero under perfect information, so that EVPI=var(α) and

(5) $$\text{EVPPI}(\phi )=\text{var}(\alpha )-E_{\phi }\left[{\text{var}}_{\alpha |\phi }(\alpha |\phi )\right]$$

(6) $$\text{EVSI}(y)=\text{var}(\alpha )-E_{y}\left[{\text{var}}_{\alpha |y}(\alpha |y)\right]$$

the expected reduction in variance given new information. Expression (5) is used by Oakley and O’Hagan (2004) and Saltelli et al. (2004) as a measure of sensitivity of the output of a deterministic model α=g(ϕ,…) to an uncertain input ϕ, termed the *main effect* of ϕ, but this has not been extended to the EVSI of potential data y in a point estimation context.

When α is a vector, the typical situation where a MPES of the form in Figure 1 is carried out, we could conduct independent VoI analyses for each component of α. In more formal decision analyses, we may want a scalar loss for the overall vector α. There are various alternatives based on generalizations v(α) of the variance, which can be used instead of the scalar variance var(α) in Equations (5) and (6) to define the expected VoI. These have been applied in the context of Bayesian study design, and we explain two examples that can be adopted for EVPPI and EVSI in our context as follows.

1. If $H=cc^{T}$ in the quadratic loss (4), for some vector of weights c, then the expected loss is $v(\alpha )=c^{T}\text{cov}(\alpha )c=\text{var}(c^{T}\alpha )$, corresponding to optimal (under squared error loss) estimation of the weighted sum of the parameters, $c^{T}\alpha$. For example, when the elements *α_s_* of α are weighted equally, the goal is to minimise the sum of all elements (*r*, *s*) of the covariance matrix, $v(\alpha )=\sum_{r,s}\text{cov}{\left(\alpha \right)}_{r,s}$, or, if the *α_s_* are also independent of each other, $v(\alpha )=\mathrm{tr}(\text{cov}(\alpha ))=\sum_{s}\text{var}(\alpha_{s})$. The same *absolute* reductions in variance for different components of α would then be valued equally. More generally, if c is given a prior, then loss (4) also arises (see Chaloner and Verdinelli 1995 and references therein). Designs that minimize (4) are Bayesian analogs of classical *A-optimal* designs. See also Lamboni, Monod, and Makowski (2011) for similar measures of sensitivity for multivariate outputs in deterministic computer models.
2. A Bayesian *D-optimal* design, on the other hand, minimizes the *determinant* v(α)=det(cov(α)) (Chaloner and Verdinelli 1995; Ryan et al. 2016). This simplifies to the *product* of the $\text{var}(\alpha_{s})$ when the *α_s_* are independent and equally weighted. Equivalently, a standardized version $\det{\left(\text{cov}(\alpha )\right)}^{1/S}$, where *S* is the number of components of α, represents a geometric average variance of the *α_s_*, adjusted for their covariance.

Here, the same *relative* reductions in variance for different components of α would then be valued equally, which would be more appropriate when the output of interest α comprises quantities on very different scales and/or with different interpretations.

### 2.4 Maximising the Expected Net Benefit of Sampling

The EVSI measures the expected *benefits* from sampling. The *costs* of sampling should also be considered. The decision-maker can then choose the design and sample size for data y to maximize the *expected net benefit of sampling* $E_{y}(b(y)-c(y))$, where b(y)=EVSI(y) is the benefit and c(y) is the cost of obtaining data y (Parmigiani and Inoue 2009). This requires benefits and costs to be measured on the same scale, which can be achieved in different ways. Improved precision of point estimates might be valued in monetary terms, as described below and illustrated in Section 5.4. Alternatively, the better knowledge given by the new data could lead to indirect benefits which could be valued, for example, improved health from better-informed health-related decision making, as discussed in Section 6. We will assume c(y) depends only on the design and sample size, thus is known in advance of observing y, so that $E_{y}(c(y))=c(y)$.

To directly translate improved precision to a monetary benefit, the decision-maker should specify the amount they are willing to pay to reduce the posterior variance by a certain amount. This willingness to pay may depend on the original posterior variance. Formally, the benefit function $b(y)=f(v_{0},v_{y})$, specified by the decision-maker, places a value on a reduction in variance (or its multivariate analog as in Section 2.3) from $v_{0}=\text{var}(\alpha )$ to $v_{y}$, the variance after collecting new data y. For example, if any *absolute* variance reduction is valued the same way (as in A-optimal design, see Section 2.3), $f(v_{0},v_{y})=\lambda (v_{0}-v_{y})$, where *λ* is the constant willingness-to-pay for one unit of variance reduction. The expected benefit is then $E_{y}(b(y))=\lambda (v_{0}-E_{y}({\text{var}}_{\alpha |y}(\alpha |y))$, which equals EVSI(y) using the quadratic loss function (4) multiplied by a constant *λ*. Alternatively, if the same *relative* gains are valued equally (as in D-optimal design), the decision-maker could specify *λ* as the amount they are willing to pay to (e.g.,) halve the variance, so that $f(v_{0},v_{y})=f(v_{0},v_{0}/2^{k})=k\lambda$, for $k=\;\text{log}\;(v_{0}/v_{y})/\;\text{log}\;(2)$.

## 3 Computation of VoI

### 3.1 Partial Perfect Information

Computation of the EVPPI in general is not straightforward. Given a sample from the posterior distribution, the first term in Equation (1) can be calculated by a Monte Carlo mean. The double expectation in the second term is more challenging. While it can be evaluated using nested Monte Carlo, this is expensive. Strong, Oakley, and Brennan (2014) proposed a method for estimating the EVPPI in the special case of finite choice decisions (Equation 3) which uses only a single Monte Carlo loop. To estimate EVPPI (1) in a broader class of decision problems, which also includes point estimation, the method needs to be generalized.

Strong, Oakley, and Brennan (2014) estimated formula (3) by expressing

(7) $$\alpha_{d}=E_{\alpha_{d}|\phi }(\alpha_{d}|\phi )+\epsilon =g_{d}(\phi )+\epsilon$$

for each d=1,…,D, where ϵ is an error term with mean zero. Then, $g_{d}(\phi )$ is estimated by regression of *α_d_* on ϕ, fitted to a Monte Carlo sample of $(\alpha_{d}^{\left(k\right)},\phi^{\left(k\right)}):k=1,\ldots ,K$. If ϕ comprises *p* parameters that could be learned simultaneously, the regression will have *p* predictors. Since the functional form of $g_{d}()$ will not be known in general, nonparametric regression methods are used. This produces a fitted value ${\hat{g}}_{d}(\phi^{\left(k\right)})$ for each *k*, which allows the second term in Equation (3) to be estimated by a Monte Carlo mean

(8) $$\begin{array}{c} E_{\phi }[\underset{{}_{d}}{\min}(E_{\theta |\phi }(\alpha_{d}))]=E_{\phi }[\underset{{}_{d}}{\min}(E_{\alpha_{d}|\phi }(\alpha_{d}|\phi ))]\\ \approx \frac{1}{K}\sum_{k=1}^{K}\underset{{}_{d}}{\min}({\hat{g}}_{d}(\phi^{\left(k\right)})). \end{array}$$

Our generalization of this approach computes EVPPI (Equation 1) in a broader class of decision problems defined as follows. Given a state of knowledge about the decision-relevant quantities α represented by a distribution ψ(·), the expected loss under the optimal decision should be a known function *h* of the mean of α under that distribution

(9) $$E_{\psi }(L(d_{\psi }^{*},\theta ))=h(E_{\psi }(\alpha )).$$

If ψ(·) is the current posterior, this is $h(E_{\alpha }(\alpha ))$, and if we were to learn the value of ϕ, the expected loss would be $h(E_{\alpha |\phi }(\alpha |\phi ))$. The method of Strong, Oakley, and Brennan (2014) only applies to the special case, where α is a vector and $h(E(\alpha ))=\min_{d}\{E(\alpha_{d})\}$. To estimate $E_{\alpha |\phi }(\alpha |\phi )$ in more general problems, we use a similar principle to (7–8), by expressing

(10) $$\alpha =E_{\alpha |\phi }(\alpha |\phi )+\epsilon =g(\phi )+\epsilon$$

then fitting a regression model g() of α on ϕ allows us to estimate

$$E_{\phi }[E_{\theta |\phi }(L(d_{\phi }^{*},\theta ))]=E_{\phi }[h(E_{\alpha |\phi }(\alpha |\phi ))]\approx \frac{1}{K}\sum_{k=1}^{K}h(\hat{g}(\phi^{\left(k\right)})).$$

Point estimation problems are also a special case of Equation (9), for example, for estimation of a scalar *α* with quadratic loss, $h(E_{\alpha }(\alpha ))=E[{\left(\alpha -E_{\alpha }(\alpha )\right)}^{2}]=\text{var}(\alpha )$. Therefore to calculate EVPPI in this case (Equation (5)), we estimate $\text{var}(\alpha |\phi^{\left(k\right)})$ by the squared residual ${\left(\alpha -\hat{g}(\phi^{\left(k\right)})\right)}^{2}$, substitute this for $h(\hat{g}(\phi^{\left(k\right)}))$ and estimate $E_{\phi }\left[{\text{var}}_{\alpha |\phi }(\alpha |\phi )\right]$ as the mean, over *k*, of the squared residuals. Equivalently, we can estimate $\text{var}(\theta )-E_{\phi }\left[{\text{var}}_{\alpha |\phi }(\alpha |\phi )\right]={\text{var}}_{\phi }(E_{\alpha |\phi }(\alpha |\phi ))$ as the variance, over *k*, of the fitted values. Similarly, for vector α and loss functions based on cov(α), we can fit regressions to get the marginal mean for each component *α_d_*, and calculate the empirical covariance matrix of the residuals.

Several methods of nonparametric regression have been suggested. For small *p*, Strong, Oakley, and Brennan (2014) used generalized additive models, with tensor products of splines to represent interactions between components of ϕ. Where ϕ included about *p* = 5 or more components, Gaussian process regression was recommended as a more efficient way of modeling interactions, though the resulting matrix computations rapidly become impractical as the MCMC sample size *K* increases. Heath, Manolopoulou, and Baio (2016) developed an integrated nested Laplace approximation for fitting Gaussian processes more efficiently where p≥2. For the application in Section 4 (with *K* = 150, 000, p≤3), we have found multivariate adaptive regression splines (Friedman 1991) via the *earth* R package (Milborrow 2011) to be more efficient. Standard errors for the EVPPI estimates can be calculated in general by simulating from the asymptotic normal distribution of the regression coefficients (Mandel 2013).

### 3.2 Sample Information

The regression method above can also be used to estimate the expected value of sample information EVSI(y). This again requires a generalization of the approach described by Strong et al. (2015) from finite decision problems to any problem satisfying condition (9), including point estimation. The method requires that the information provided by the data y can be expressed as a low-dimensional sufficient statistic T(y), so that $E_{\alpha |y}(\alpha |y)=E_{\alpha |y}(\alpha |T(y))$. This could be a point estimator of the parameter *μ* (as in Figure 1) that y gives direct information on. As in (10), we can write

$$\alpha =E_{\alpha |y}(\alpha |T(y))+\epsilon =g(T(y))+\epsilon$$

and estimate g() using a regression fitted to a Monte Carlo sample of $(\alpha^{\left(k\right)},T(y^{\left(k\right)})):k=1,\ldots ,K$, where $y^{\left(k\right)}$ are drawn from their posterior predictive distribution. Then, the fitted values $\hat{g}(T(y^{\left(k\right)}))$ enable the double expectation to be estimated as

$$E_{y}[E_{\theta |y}(L(d_{y}^{*},\theta ))]=E_{y}[h(E_{\alpha |y}(\alpha |y))]\approx \frac{1}{K}\sum_{k=1}^{K}h(\hat{g}(T(y^{\left(k\right)}))).$$

Then, for example, for point estimation with quadratic loss, this is the estimated residual variance from the regression, as in Section 3.1.

## 4 The HIV Prevalence MPES Model

We consider the submodel of the full HIV burden model (De Angelis et al. 2014; Kirwan et al. 2016) that estimates HIV prevalence in men who have sex with men (MSM), in London. We define three subgroups of MSM: those who have attended a genitourinary medicine (GUM) clinic in the past year (GMSM), those who have not (NGMSM), and previous MSM (PMSM), men who no longer have sex with men. We denote the proportion of all men who are in these subgroups by *ρ_G_*, *ρ_N_*, and *ρ_P_* respectively. For each group g∈(G,N,P), we aim to estimate simultaneously these subgroup proportions *ρ_g_*, prevalence of HIV in this group *π_g_* and the proportion of infections that are diagnosed, *δ_g_*. Given these parameters, further important quantities are easily derived: the prevalence of diagnosed ($\pi_{g}\delta_{g}={\left(\pi \delta \right)}_{g}$) and undiagnosed ($\pi_{g}(1-\delta_{g})={\bar{\left(\pi \delta \right)}}_{g}$) infection; and the numbers of MSM living with diagnosed ($\mu_{\mathrm{Dg}}=\mu_{\text{pop}}\rho_{g}{\left(\pi \delta \right)}_{g}$) and undiagnosed ($\mu_{\mathrm{Ug}}=\mu_{\text{pop}}\rho_{g}{\bar{\left(\pi \delta \right)}}_{g}$) infection, where $\mu_{\text{pop}}$ is the number of men (MSM and non-MSM) living in London. Since the prevalence among PMSM is much lower, this subgroup is not examined in detail.

**Fig. 3:**
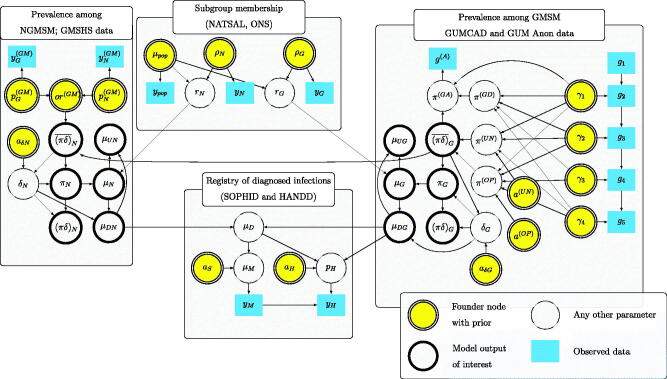
Directed acyclic graph for HIV prevalence estimation model.

We construct a Bayesian model to link the unknown $\rho_{g},\pi_{g},\delta_{g}$ with the available evidence provided by various routinely-collected and survey datasets as well as expert belief. Figure 3 shows a directed acyclic graph representing this model, in the form of Figure 1, distinguishing founder nodes, observed data, and outputs of interest. The following sections explain in detail the quantities and relationships illustrated in Figure 3. All data and estimates refer to the year 2012 (unless indicated) and the Greater London area.

### 4.1 Subgroup Membership

The total male population of London, $\mu_{\text{pop}}$, is informed by published data $y_{\text{pop}}$ (Office for National Statistics 2012), assumed to be a Poisson count: $y_{\text{pop}}∼\mathrm{Po}(\mu_{\text{pop}})$. A log-normal prior for $\mu_{\text{pop}}$ is assumed, $\text{log}\;(\mu_{\text{pop}})∼N(0,1000^{2})$. The number of people in each group *g* is estimated as $r_{g}=\rho_{g}\mu_{\text{pop}}$. Estimates of the subgroup proportions *ρ_g_* are informed by data from the National Survey of Sexual Attitudes and Lifestyles (Mercer et al. 2013): *y_G_* = 7, $y_{N}=38,y_{P}=10$, out of $y_{\text{NAT}}=824$ men, which we assume to come from a multinomial distribution with probabilities $\rho_{G},\rho_{N},\rho_{P}$ given a uniform Dirichlet prior. Thus, the expected number of people with HIV (diagnosed or undiagnosed) in group *g* is $\mu_{g}=\pi_{g}r_{g}$.

### 4.2 Registry of Diagnosed Infections and Diagnosed Prevalence

Individuals diagnosed with HIV and accessing care in the UK are reported to the HIV and AIDS Reporting System (Kirwan et al. 2016). From the 2012 version of this dataset, known as SOPHID (Surveillance of Prevalent HIV Infections Diagnosed), we obtain the reported number of HIV diagnoses for MSM, $y_{M}∼\mathrm{Po}(\mu_{M})$, with *y_M_* = 8390. A reporting bias of unknown direction is assumed, through $\text{log}\;(\mu_{M})=a_{S}+\;\text{log}\;(\mu_{D})$ where $\text{exp}\;(a_{S})∼N(1,0.018^{2})$, giving a prior 90% interval of about (−3%,3%) for the adjustment to the number of MSM HIV diagnoses *μ_M_*. After adjustment, $\mu_{D}=\mu_{\mathrm{DG}}+\mu_{\mathrm{DN}}+\mu_{\mathrm{DP}}$ is the expected number of diagnoses among MSM, summed from the expected numbers of diagnoses among GMSM, NGMSM, and PMSM, respectively. The following sections explain where $\mu_{\mathrm{DG}},\mu_{\mathrm{DN}}$ come from; *μ_DP_* is modeled using similar techniques.

Since SOPHID did not record GUM clinic attendance, to strengthen the evidence on diagnosed prevalence in GMSM, we include data from the HIV and AIDS New Diagnoses and Deaths Database (HANDD) (Kirwan et al. 2016), recording how many of the *y_M_* prevalent diagnosed MSM were newly diagnosed in 2012 and reported to have been diagnosed initially in a GUM clinic. These new diagnoses, *y_H_* = 630, are modeled as $y_{H}∼\text{Bin}(y_{M},p_{H})$, where *p_H_* is assumed to be a lower bound for the proportion of prevalent diagnosed MSM who have attended a GUM clinic in 2012. This bound is expressed through $p_{H}=a_{H}\mu_{\mathrm{DG}}/\mu_{D}$, where $a_{H}∼U(0,1)$ is the unknown probability that a prevalent diagnosed MSM who has attended a GUM clinic in 2012 was newly diagnosed that year. *y_H_* therefore gives us additional indirect information on *μ_DG_*, the number of prevalent diagnosed GMSM.

The number of *diagnosed* infections is related to the total number of infections in each group *g* as $\mu_{\mathrm{Dg}}=\delta_{g}\mu_{g}$. The proportion of infections that are diagnosed *δ_g_* is not known, but given our inferences about the undiagnosed prevalence ${\bar{\left(\pi \delta \right)}}_{g}=\pi_{g}(1-\delta_{g})$ (explained in the subsequent sections), we can exploit the implicit constraint $1-\delta_{g}>{\bar{\left(\pi \delta \right)}}_{g}$. Therefore, we define $\delta_{g}=a_{\delta g}(1-{\bar{\left(\pi \delta \right)}}_{g})$, with $a_{\delta g}∼U(0,1)$, and the diagnosed prevalence ${\left(\pi \delta \right)}_{g}=\pi_{g}\delta_{g}$ in each group follows.

### 4.3 Undiagnosed Prevalence Among GMSM

Information about undiagnosed infections in GMSM is obtained from GUMCAD (Genitourinary Medicine Clinic Activity Dataset) (Kirwan et al. 2016) a registry of attendance episodes in GUM clinics. HIV tests are offered routinely to previously undiagnosed patients. Thus, we have a sequence of observations *g_i_*, representing firstly the number of GUM clinic visitors ($g_{1}=35,121$) and then the number of patients with no previous HIV diagnosis ($g_{2}=34,187$), HIV tests offered ($g_{3}=30,570$), HIV tests accepted ($g_{4}=29,529$), and HIV diagnoses made ($g_{5}=855$). For $i=2,\ldots ,5,g_{i}∼\text{Bin}(g_{i-1},\gamma_{i-1})$, with priors $\gamma_{1},\gamma_{2},\gamma_{3}∼U(0,1)$ and $\gamma_{4}∼U(0,0.15)$ (see below). An HIV infection may therefore remain undiagnosed if either a test is not offered or the patient opts out of testing. We can then decompose the prevalence of undiagnosed infection ${\bar{\left(\pi \delta \right)}}_{G}$ into “unoffered” $\pi^{\left(\text{UN}\right)}$ and “opt-out” $\pi^{\left(\text{OP}\right)}$ components.

(11) $${\bar{\left(\pi \delta \right)}}_{G}=\pi^{\left(\text{UN}\right)}+\pi^{\left(\text{OP}\right)}.$$

Both of those require strong prior assumptions to estimate, which will later be relaxed in a sensitivity analysis (Section 4.5). First, the prevalence of infection that remains undiagnosed due to an unoffered test is

$$\pi^{\left(\text{UN}\right)}=\gamma_{1}(1-\gamma_{2})p^{\left(\text{UN}\right)},$$

where $\gamma_{1}(1-\gamma_{2})$ is the proportion of clinic attenders that are undiagnosed but not offered a test, and $p^{\left(\text{UN}\right)}$ is the probability that a test would be positive for these people. We assume the prevalence in this group is between 0.5 and 1.5 times the prevalence in people actually tested, and $\text{logit}(p^{\left(\text{UN}\right)})=\text{logit}(\gamma_{4})+a^{\left(\text{UN}\right)}$, with $a^{\left(\text{UN}\right)}∼U(\;\text{log}\;(0.5),\;\text{log}\;(1.5))$

Secondly, the prevalence of infection remaining undiagnosed due to refusing a test is

$$\pi^{\left(\text{OP}\right)}=\gamma_{1}\gamma_{2}(1-\gamma_{3})(\gamma_{4}+a^{\left(\text{EX}\right)})$$

where $\gamma_{1}\gamma_{2}(1-\gamma_{3})$ is the proportion of clinic attenders that are undiagnosed and offered a test but opt out. We assume this group has an underlying HIV prevalence higher than those given tests, but not more than 15%, so that the excess prevalence in this group is $a^{\left(\text{EX}\right)}=a^{\left(\text{OP}\right)}(0.15-\gamma_{4})$, where $a^{\left(\text{OP}\right)}∼U(0,1)$, and the prior on *γ*_4_ is truncated above at 0.15.

A small amount of additional evidence on ${\bar{\left(\pi \delta \right)}}_{G}$ is available from another dataset, GUM Anon (Public Health England, London 2012), a convenience survey of men not previously diagnosed with HIV who had attended a GUM clinic in the previous year. This gives direct information about the prevalence of HIV among previously undiagnosed GMSM,

(12) $$\pi^{\left(\text{GA}\right)}=({\bar{\left(\pi \delta \right)}}_{G}+\pi^{\left(\text{GD}\right)})/\gamma_{1},$$

where $\pi^{\left(\text{GD}\right)}=\prod_{1}^{4}\gamma_{r}$ is the prevalence of newly diagnosed infection among clinic attenders. The data in GUM Anon are $g^{\left(A\right)}∼\text{Bin}(g^{\left(\text{AN}\right)},\pi^{\left(\text{GA}\right)})$, where $g^{\left(A\right)}=4$ and $g^{\left(\text{AN}\right)}=85$.

### 4.4 Undiagnosed Prevalence Among NGMSM

To inform undiagnosed HIV prevalence in NGMSM, we use data from the Gay Men’s Sexual Health Survey (GMSHS) (Aghaizu et al. 2016), based on face-to-face interviews in selected venues where participants were offered anonymous HIV tests. While this group is likely to have a higher HIV prevalence than the general population of MSM, it is assumed that the *relative odds* of having HIV between NGMSM and GMSM is the same as in the general population. The GMSHS data provide the numbers $y_{g}^{\left(\text{GM}\right)}$ out of $n_{g}^{\left(\text{GM}\right)}$ previously undiagnosed people in group *g* who tested positive for HIV (20 out of 493 GMSM and 20 out of 452 NGMSM) so that $y_{g}^{\left(\text{GM}\right)}∼\text{Bin}(n_{g}^{\left(\text{GM}\right)},p_{g}^{\left(\text{GM}\right)})$, with $p_{g}^{\left(\text{GM}\right)}∼U(0,1)$. Defining the odds o(p)=p/(1−p), we apply the resulting odds ratio $or^{\left(\text{GM}\right)}=o(p_{N}^{\left(\text{GM}\right)})/o(p_{G}^{\left(\text{GM}\right)})$ to the baseline estimated from GUMCAD (Section 4.3), giving $o({\bar{\left(\pi \delta \right)}}_{N})$ = $o({\bar{\left(\pi \delta \right)}}_{G})or^{\left(\text{GM}\right)}$.

### 4.5 Alternative Assumptions

The results presented in Section 5 are for the above model assumptions, unless specified otherwise. Two alternative assumptions are also explored.

- Undiagnosed prevalence from GUM Anon only
  To avoid the strong prior assumptions on prevalence among those not offered a test or refusing a test, which are necessary to use the GUMCAD data to infer ${\bar{\left(\pi \delta \right)}}_{g}$, we could infer ${\bar{\left(\pi \delta \right)}}_{g}$ from GUM Anon alone. To construct this model, we replace Equation (11) by a *U*(0, 1) prior on ${\bar{\left(\pi \delta \right)}}_{g}$, although the GUMCAD data are still used to estimate the parameters $\pi^{\left(\mathrm{GD}\right)}$ and *γ*_1_ relating the prevalence in GUM Anon to ${\bar{\left(\pi \delta \right)}}_{g}$.
- GUMCAD also informs diagnosed prevalence
  Instead of being inferred indirectly through the graph, the diagnosed prevalence can be modeled directly as
  (13) $${\left(\pi \delta \right)}_{G}=(1-\gamma_{1})+\gamma_{1}\gamma_{2}\gamma_{3}\gamma_{4},$$

where $1-\gamma_{1}$ is the probability of a previous diagnosis, and $\gamma_{1}\gamma_{2}\gamma_{3}\gamma_{4}$ is the probability of a new diagnosis, in GUMCAD. This is not done in the base case due to concerns about inconsistencies in reporting between GUMCAD and SOPHID/HANDD.

## 5 VoI Results in the HIV Model

**Fig. 4:**
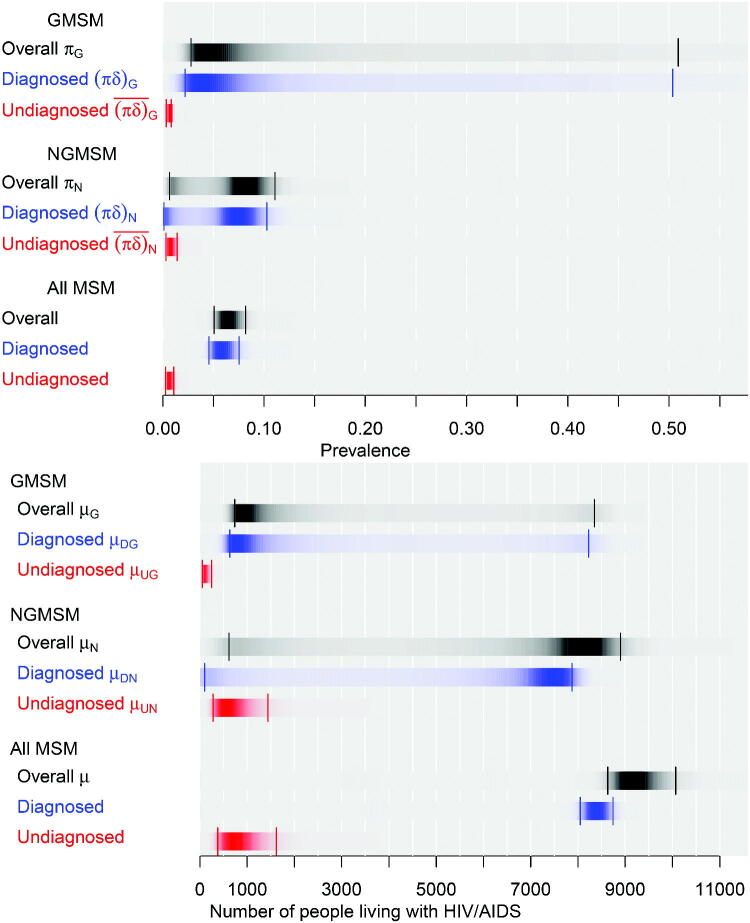
Posterior distributions of HIV prevalence (top) and numbers of MSM living with HIV/AIDS (bottom), London 2012. Darkness within each strip proportional to posterior density, with 95% credible intervals indicated.

The model outputs of interest (as in Figures 1, 3) are $\alpha =({\left(\pi \delta \right)}_{G},{\left(\pi \delta \right)}_{N},{\bar{\left(\pi \delta \right)}}_{G},{\bar{\left(\pi \delta \right)}}_{N},\mu_{\text{DG}},\mu_{\text{DN}},\mu_{\text{UG}},\mu_{\text{UN}},\mu$), the diagnosed and undiagnosed prevalences among both GMSM and NGMSM, and the corresponding absolute numbers of people living with HIV (or “case-counts”), and the total number of MSM with HIV $\mu =\mu_{\text{DG}}+\mu_{\text{DN}}+\mu_{\text{UG}}+\mu_{\text{UN}}$. Samples from the posterior are generated using Hamiltonian Monte Carlo methods in the Stan software (Stan Development Team 2016). These are illustrated in Figure 4 along with the overall prevalence $\pi_{g}={\left(\pi \delta \right)}_{g}+{\bar{\left(\pi \delta \right)}}_{g}$ in each group *g*, and each of these quantities summed over the two groups *g*. The estimates of diagnosed prevalence in all MSM (top panel) are reasonably precise, while the corresponding estimates for NGMSM and GMSM are more uncertain. Estimates of undiagnosed prevalence are lower and more precise. Full results under the two alternative assumptions are presented in the supplementary figures.

### 5.1 Partial Perfect Information (EVPPI) for Single Outputs

Defining the decision problem as point estimation of α with quadratic loss, we use EVPPI formula (5) to determine which parameters ϕ contribute most to the uncertainty about each component of α, thus which ϕ may be worth learning more precisely. We will take ϕ to include the founder nodes of the graph illustrated in Figure 3. Since they are related to the α through a network of deterministic functions, perfect knowledge of these implies perfect knowledge of α. Each of the ϕ are either directly informed by data or given a substantive prior distribution based on belief. In the former case, EVPPI measures the maximum potential value of collecting more data from the same source. In the latter case, it will not necessarily be feasible to collect data to improve the precision of the belief, but EVPPI is still useful as a measure of how much of the uncertainty in α is explained by the uncertainty in the parameter.

**Fig. 5:**
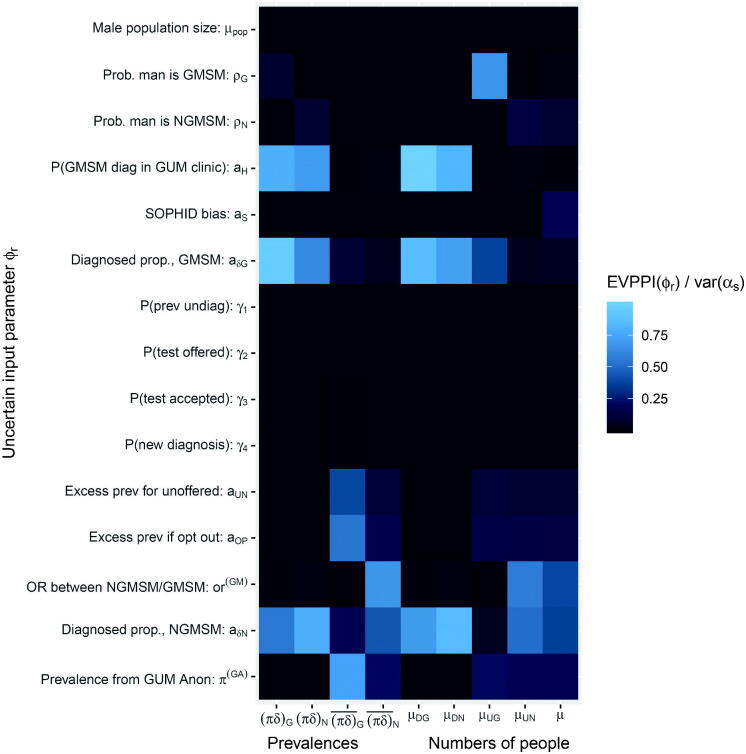
Expected value of partial perfect information in the HIV prevalence model.

The results are presented in Figure 5 as a grid whose *r*, *s* entry is colored according to ${\text{EVPPI}}_{\alpha_{s}}(\phi_{r})/\text{var}(\alpha_{s})$, the proportion of variance in *α_s_* which would be reduced if we learnt $\phi_{r}$. The lighter cells correspond to $\phi_{r}$ with greater EVPPI. Standard errors in these and all following EVPPI and EVSI estimates, arising from uncertainty in the coefficients of the regression (10), were negligible, at less than 1% of the EVPPI or EVSI estimates.

The parameters $a_{\delta G}$ and $a_{\delta N}$, governing the proportions of HIV infections that are diagnosed in each of the two groups, and the probability *a_H_* that a GMSM is newly diagnosed in a GUM clinic, explain most of the uncertainty in the *diagnosed* prevalences ${\left(\pi \delta \right)}_{G},{\left(\pi \delta \right)}_{N}$ and the corresponding numbers of people diagnosed $\mu_{\text{DG}},\mu_{\text{DN}}$. Direct data on any of these parameters would be difficult to obtain. However, if we were willing to make the assumption in Equation (13), the estimates of diagnosed prevalence would become more precise, for example the posterior median (SD) of ${\left(\pi \delta \right)}_{G}$ would change from 0.06 (0.13) to 0.051 (0.001), though the extent of uncertainty around ${\left(\pi \delta \right)}_{N},\mu_{\mathrm{DN}}$ would not change substantively.

For the *undiagnosed* prevalences ${\bar{\left(\pi \delta \right)}}_{G},{\bar{\left(\pi \delta \right)}}_{N}$ and undiagnosed case count $\mu_{\text{UG}}$, Figure 5 shows that more GUM-Anon data (via $\pi^{\left(GA\right)}$), more GMSHS data (via $or^{\left(GM\right)}$) and more NATSAL data (via $\rho_{\text{UG}}$), respectively, would give the greatest uncertainty reductions. These outcomes, however, are already precisely estimated in absolute terms (Figure 4). The number of NGMSM $\mu_{\text{UN}}$ with undiagnosed HIV is more uncertain, with 95% CrI (279,1442), and more GMSHS data would be potentially valuable to reduce this uncertainty.

If ${\bar{\left(\pi \delta \right)}}_{G}$ were informed only from the 4 infections out of 85 people observed in GUM Anon (alternative assumption (a)), the estimates of undiagnosed prevalence or case counts become extremely uncertain, for example, $\text{var}(\mu_{\text{UN}})$ increases from 302^2^ to 2872^2^. We could reduce this uncertainty by collecting more GUM Anon data—since ${\text{EVPPI}}_{\mu_{\text{UN}}}(\pi^{\left(\text{GA}\right)})$ is p=62% of $\text{var}(\mu_{\text{UN}})$, more GUM Anon data could reduce $\text{var}(\mu_{\text{UN}})$ to a minimum of $2872^{2}(1-p)=1777^{2}$ (note that the square root of the expected variance after learning data is not the same as the expected standard deviation).

### 5.2 Partial Perfect Information for Multiple Outputs

Staying with alternative assumption (a), suppose we wish to calculate the maximum potential value of extra GUM Anon data for *jointly* reducing the uncertainty about the number of GMSM, NGMSM, and PMSM with undiagnosed HIV, so that α is the vector $\left(\mu_{\text{UG}},\mu_{\text{UN}},\mu_{\text{UP}}\right)$. As described in Section 2.3, we could simply calculate the standard EVPPI based on a scalar output *α* redefined as their sum, $\mu_{U}=\mu_{\text{UG}}+\mu_{\text{UN}}+\mu_{\text{UP}}$, the total number of MSM with undiagnosed HIV, whose posterior median is 5149 (SD 3280). This would ensure that any data expected to reduce the variance of any of these three outputs by the same (additive) amount would be valued equally. From this, we find that extra GUM Anon data would be expected to reduce $\text{var}(\mu_{U})$ from 3280^2^ to a minimum of 1801^2^. Since *μ_U_* is dominated by NGMSM (posterior median of $\mu_{\text{UN}}$ is 4185), this is mostly explained by an expected reduction in $\text{var}(\mu_{\text{UN}})$ from 2864^2^ to a minimum of 1770^2^.

Alternatively, suppose both the prevalences and the case counts are of interest, for example in NGMSM, so that $\alpha =({\bar{\left(\pi \delta \right)}}_{N},\mu_{\text{UN}})$. Since these two components are on very different scales, the Bayesian “D-optimality” criterion v(α)=det(cov(α)) would be a preferable measure of overall expected loss due to uncertainty. We use this criterion to compare the maximum expected value of extra GUM Anon data and extra GMSHS data, which combine to estimate the outcomes for NGMSM as described in Section 4.4. The EVPPI is interpreted as the expected reduction in the product of $\text{var}({\bar{\left(\pi \delta \right)}}_{N})$ and $\text{var}(\mu_{\text{UN}})$ given by extra GUM Anon or GMSHS data, adjusted for their covariance. This is 425 and 135, respectively, favoring extra data from GUM Anon. Though in this example, examining expected reductions in $\text{var}({\bar{\left(\pi \delta \right)}}_{N})$ or $\text{var}(\mu_{\text{UN}})$ separately would lead to the same conclusion, since ${\bar{\left(\pi \delta \right)}}_{N}$ is defined as the proportion $\mu_{\text{UN}}/r_{N}$ of NGMSM with HIV, and GUM Anon and GMSHS are not informative about the number *r_N_* of NGMSM, thus extra data informs $\mu_{\text{UN}}$ entirely through information on ${\bar{\left(\pi \delta \right)}}_{N}$ (or vice versa).

### 5.3 Sample Information (EVSI)

We now estimate the expected value of data with specific sample sizes for improving the precision of the estimated number of people *μ_U_* with undiagnosed HIV. Using the GUMCAD data and associated strong prior assumptions, the posterior median of *μ_U_* is 804 (SD 320), compared to 5149 (SD 3280) with this information excluded (a). We compare the value of additional data from GUM Anon and additional data from GMSHS (on top of their original sample sizes of 85 and 945, respectively) for reducing these posterior standard deviations.

The EVSI is computed for a series of sample sizes *n* using the method in Section 3.2. For GUM Anon (Section 4.3), the sufficient statistic T(y) consists of the empirical HIV prevalence y/n from an additional survey $y∼\text{Bin}(n,\pi^{\left(\text{GA}\right)})$. For GMSHS (Section 4.4), given a sample size *n*, $y=(N_{G}^{\left(GM\right)},Y_{G}^{\left(GM\right)},Y_{N}^{\left(GM\right)})$, where $N_{G}^{\left(\text{GM}\right)}$ is the number of previously undiagnosed MSM in the future sample of *n* who attend GUM clinics (the equivalent of the observed $n_{G}^{\left(\text{GM}\right)}=493$). Then $Y_{G}^{\left(\text{GM}\right)}$ and $Y_{N}^{\left(\text{GM}\right)}$ are the numbers of men out of denominators $N_{G}^{\left(\text{GM}\right)}$ and $N_{N}^{\left(\text{GM}\right)}=n-N_{G}^{\left(\text{GM}\right)}$ (GMSM and NGMSM, respectively) who test positive for HIV, the equivalents of the observed $y_{G}^{\left(GM\right)}=20,y_{N}^{\left(GM\right)}=492$. We take $T(y)=o({\hat{p}}_{N}^{\left(GM\right)}(y))/o({\hat{p}}_{G}^{\left(GM\right)}(y))$, a point estimator of the odds ratio, where ${\hat{p}}_{G}^{\left(GM\right)}(y)$ is an estimator of the proportion of MSM in group *g* who have HIV. To avoid zeros in the denominator $o({\hat{p}}_{G}^{\left(\text{GM}\right)}(y))$, we use a Bayesian estimator ${\hat{p}}_{G}^{\left(\text{GM}\right)}(y)=(Y_{G}^{\left(\text{GM}\right)}+0.5)/(N_{G}^{\left(\text{GM}\right)}+1)$, the posterior mean of a binomial proportion under a Jeffreys Beta(0.5,0.5) prior, rather than the empirical proportion $Y_{G}^{\left(\text{GM}\right)}/N_{G}^{\left(\text{GM}\right)}$.

**Fig. 6:**
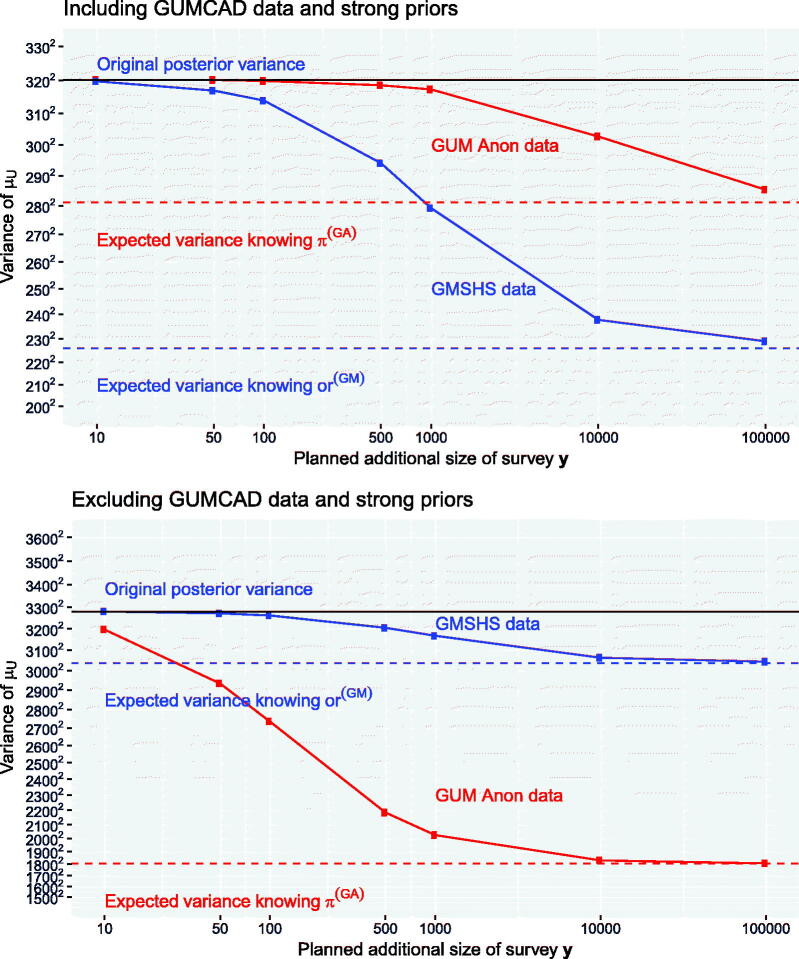
EVSI: value of additional data from GUM Anon or GMSHS for reducing the variance of the total number of MSM with undiagnosed HIV, $\mu_{U}=\mu_{\text{UG}}+\mu_{\text{UN}}$. The *x*-axis is on the log scale. The *y*-axis is the variance, with the labels as SD^2^.

Figure 6 shows $\text{var}(\mu_{U})-\mathrm{EVSI}(y)$, the expected variance remaining after data collection, under the two alternative assumptions. With the strong priors, *μ_U_* is relatively well informed, and extra data from GUM Anon at realistic sample sizes (1000 or less) would not noticeably reduce $\text{var}(\mu_{U})$. GMSHS data would be more valuable, through improving the estimate of $\mu_{\text{UN}}$, the more uncertain contributor to $\mu_{U}=\mu_{\text{UG}}+\mu_{\text{UN}}$. 1000 extra observations from GMSHS would be expected to reduce $\text{var}(\mu_{U})$ from 320^2^ to 279^2^.

Without the strong prior information, $\text{var}(\mu_{U})=3280^{2}$ is substantially greater, and *μ_U_* is only directly informed by the 85 observations from GUM Anon. Extra data from this source would be valuable, for example, another 500 observations would be expected to reduce this variance to 2184^2^. Relative to these improvements, GMSHS data of the same size would be much less valuable. GMSHS data, however, would be expected to give around the same *absolute* reductions in $\text{var}(\mu_{U})$, whether or not the strong priors are included.

### 5.4 Net Benefit of Sampling

**Fig. 7:**
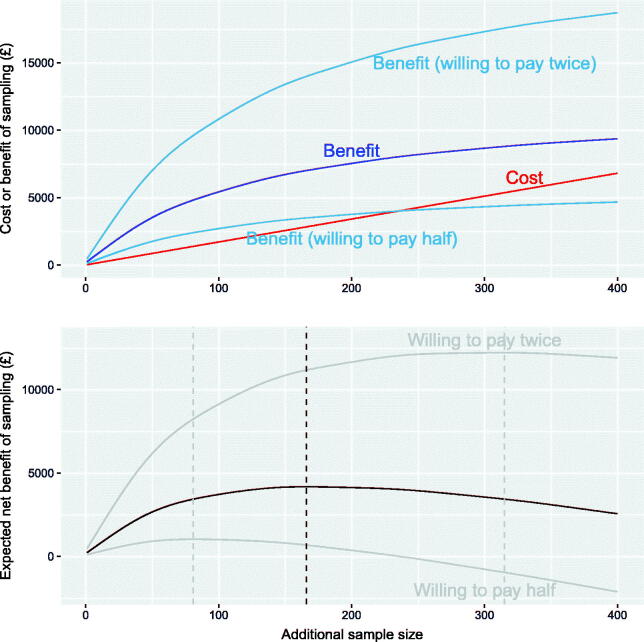
Expected cost, benefit, and net benefit of sampling up to 400 extra participants from GUM Anon, if we wish to reduce the variance of *μ_U_*, the number of MSM with undiagnosed HIV. The optimal sample size is illustrated as a dotted line.

The benefits from improved precision of estimates of *μ_U_* must be traded off with the costs of data collection, to determine an optimal sample size for extra survey data. Consider the scenario which excluded the GUMCAD data and associated strong priors. In the GUM Anon survey, there was a cost of around £17 per participant, which is assumed to be the same for collecting further data from this source. The cost c(y) is illustrated against sample sizes of y from 1 to 400 by the straight line in Figure 7. Suppose also that the decision-maker is willing to pay £5000 to reduce the variance of *μ_U_* by $\mathrm{dv}=3271^{2}-2771^{2}$, which in this case would reduce the standard deviation by 500, from 3271 to 2771. The willingness to pay per unit variance is then λ=5000/dv.

Collecting extra data y will give an expected reduction in $\text{var}(\mu_{U})$ of EVSI(y), as illustrated in Figure 7. The resulting expected (monetary) benefit $E_{y}(b(y))$ (Section 2.4) is shown to be a nonlinear function of the sample size of y, with an asymptote representing the expected value of partial perfect information on $\pi^{\left(\text{GA}\right)}$. Hence, the expected *net benefit* of sampling $E_{y}(b(y)-c(y))$ is illustrated in the bottom panel of Figure 7. The expected benefits of sampling always exceed the costs, and the net benefit is maximized at a sample size of 166. Also illustrated are the benefit and net benefit that would result if the decision maker was willing to pay twice or half the original amount, 2λ or λ/2. The corresponding optimal sample sizes would be 315 or 81, respectively.

## 6 Summary and Potential Further Work

We have presented tools to find the most influential sources of uncertainty in MPES models and determine the expected value of extra data. We generalized methods, previously applied only in deterministic models, to complex graphical models, a class which also includes hierarchical models. We have shown how VoI methods developed for formal finite-choice decision problems can be extended to deal with estimation of single or multiple quantities.

While the purpose of our model was to estimate a quantity of interest to policy-makers, the same methods could be used for models to compare specific health policies. Sections 2.4 and 5.4 illustrated how the benefits from more precise estimates of HIV prevalence could be converted directly into a monetary value. An alternative approach would be to value the indirect health gains that would result from better data, through better-informed health policies. This would allow standard health economic principles to be used (see, e.g., Briggs, Sculpher, and Claxton 2006). For example, the National Institute for Health and Care Excellence in the UK recommends that a new health-care intervention is funded by the National Health Service if the cost per quality-adjusted life year (QALY) gained, compared to current practice, is less than around λ=£20,000, implying a willingness to pay of *λ* per QALY. This is a choice between two actions d∈ “{ accept, reject } intervention” (as in Section 2.3) with loss $L(d,\theta )=c_{d}(\theta )-\lambda q_{d}(\theta )$, where $q_{d}(\theta )$ is the expected QALY and $c_{d}(\theta )$ is the expected cost for a person under action *d*, from a health economic model with parameters *θ*. See, for example, Carmona, O’Rourke, and Robinson (2016); Baggaley et al. (2017), for how such models might be built for HIV testing interventions to increase the proportion *δ* of people who are diagnosed. Briefly, any QALY gains strongly depend on the underlying prevalence of HIV among the population receiving the intervention. Thus, improved estimates of prevalence will lead to more precise estimates of the QALY gains, and a better-informed decision about whether to implement the intervention, which may result in a better use of health service resources. VoI methods may then be used to decide whether further information should be collected to support the decision.

In the HIV application, we found that structural assumptions, such as whether to include a particular piece of information, were influential to both the parameter estimates and the VoI. Such uncertainties might be parameterized (see, e.g., Strong, Oakley, and Chilcott 2012), for example a particular prior or dataset of uncertain relevance could be discounted using an unknown weight (e.g., Neuenschwander, Branson, and Spiegelhalter 2009). The EVPPI of the extra parameter would then quantify this uncertainty in the context of all other uncertainties, referred to as the “expected value of model improvement” by Strong and Oakley (2014).

Note that VoI refers to the expected value of *potential future* information, which differs from the *observed* value of a dataset *x_i_ currently* included in the model. The latter could be computed as the observed reduction in loss when the model is refitted without *x_i_*. This could demonstrate the value of past data to the policymaker responsible for funding the collection of future data of the same type. For surveys or longitudinal studies conducted at regular intervals, VoI might be used to determine the expected value of future surveys or follow-up, although a full analysis would require modeling the expected changes through time in the quantities, such as disease prevalence or incidence, informed by the data.

While our method is broadly applicable, the details of computation for different decision problems and loss functions may be different. We discussed finite-action decisions and point estimation. A more general decision problem is to estimate the entire uncertainty distribution of θ. The standard posterior p(θ|y) is then optimal under a log scoring rule (Bernardo and Smith 1994), and (following Lindley 1956) standard Bayesian design theory aims to maximize the information gain from new data y, which we can write as $\text{EVSI}(y)=E_{\theta }(-\;\text{log}\;(p(\theta )))+E_{y}E_{\theta |y}\{\;\text{log}\;(p(\theta |y))$. Under linear models (Chaloner and Verdinelli 1995), this is equivalent to minimising det(cov(θ)), but more generally this is challenging to compute (Ryan et al. 2016).

Note that the VoI approach to sensitivity analysis is an example of the “global” approach, which examines the changes in model outputs given by varying parameters within the ranges of their belief distributions. The “local” approach is based on examining the posterior geometry resulting from small parameter perturbations around a base case, for example, Roos et al. (2015) assess the robustness of hierarchical models to prior assumptions in this way. While the global approach is easier to interpret, as discussed by Oakley and O’Hagan (2004) and Roos et al. (2015), it conditions on one particular prior specification, and parameterising all potential prior beliefs or structural assumptions would be impractical.

The regression method for VoI computation that we described requires only a MCMC sample from the joint distribution of parameters of interest ϕ and outputs α. Additionally for EVSI it requires that the information in the new data y can be condensed into an analytic sufficient statistic T(y). Alternative methods which exploit particular analytic structures of g(), where *α* is a known function g(ϕ), thus avoiding a regression approximation, were discussed by Madan et al. (2014) for EVPPI and Ades et al. (2004) for EVSI. Menzies (2016) also presented an importance resampling method for EVSI computation which needs only a single MCMC sample and not a sufficient statistic.

In conclusion, the consideration of future evidence requirements is an often-neglected part of statistical analysis. The VoI methods we have presented provide a practicable set of tools for achieving this aim in the context of Bayesian evidence synthesis.

## Supplementary Materials

A supplementary document provides estimates of HIV prevalence and expected value of partial perfect information under the alternative assumptions described in Section 4.5.

## Supplementary Material

Supplemental Material
