# Supplementary material for: Value of Information: Sensitivity Analysis and Research Design in Bayesian Evidence Synthesis
Source: J Am Stat Assoc. 2019 Apr 30;114(528):1436–49. doi: 10.1080/01621459.2018.1562932 (PMC7034331; doi:10.1080/01621459.2018.1562932)
Supplement: Supplemental Material [file UASA_A_1562932_SM9181.zip › UASA_A_1562932_Supplement/JASA_ACS_author_form.pdf]

JASA ACS Reproducibility Initiative - Author Contributions Checklist Form

Reproducibility information for the article “Value of Information: Sensitivity Analysis and Research Design in Bayesian Evidence Synthesis”

Data

Aggregate data from NATSAL, SOPHID, HANDD, ONS, GUMCAD, GUM Anon and GMSHS, as described in Section 4 of the manuscript.

The individual-level data underlying the NATSAL data used here are publicly available from http://www.natsal.ac.uk/natsal-3.aspx. Public Health England have given permission for the other aggregate datasets to be made available with this article – the underlying individual-level data sources are the property of Public Health England and not publicly available.

The aggregate data are given and explained in full in the text of Section 4 of the manuscript, and also available in R format from https://github.com/chjackson/voibayes, file **R/mpesdata.rda**.

Code

Code for Bayesian evidence synthesis and Value of Information calculations, to reproduce the results in the manuscript, is available from https://github.com/chjackson/voibayes. Commit reference 9fbd510 at the time of submission. This includes the following files

voibayes.Rnw, voibayes-extraplots.Rnw : paper manuscript and supplementary figures, in LaTeX with embedded R code to generate the results and plots in the paper. The paper can be “compiled” to regenerate the results and plots using the R package **knitr**. This depends on the following files, supplied in the “**R**” directory of the repository.

MCMC output samples, in R format:

- **sam.rda**: (base case)
- **samnogu.rda**: alternative assumption (a): Undiagnosed prevalence from GUM Anon only
- **samgudnd.rda**: alternative assumption (b): GUMCAD also informs diagnosed prevalence

R code to generate results from these samples

- **plots.r**: posterior summaries (text and Figure 4)
- **evppi.r**: expected value of partial perfect information (text and Figure 5)
- **evsi.r:** expected value of sample information (text and Figure 6)

Optionally, the MCMC output samples can be regenerated using the Stan software (through the **rstan** R package***)*** by running the following file

- **mpes.r**

which depends on the Stan (http://mc-stan.org) model files

- **mpes.stan,**
- **mpes-nogu.stan** (assumption (a)),
- **mpes-gudnd.stan** (assumption (b))

and the data in

- **mpesdata.rda**

Each Stan model fit took about 8 minutes, using 3 cores of a typical desktop computer.

The analysis and presentation used R version 3.4.4 (2018-03-15), and the following R CRAN package versions

"knitr" "1.20"
"earth" "4.6.3"
"denstrip" "1.5.4"
"rstan" "2.18.2" (which includes the Stan libraries)
"ggplot2" "3.1.0"
"mgcv" "1.8-25"
"gridExtra" "2.3"

Instructions for Use

To reproduce the whole manuscript, run

library(knitr)
knit2pdf("voibayes.Rnw")
