# Supplementary material for: Value of Information: Sensitivity Analysis and Research Design in Bayesian Evidence Synthesis
Source: J Am Stat Assoc. 2019 Apr 30;114(528):1436–49. doi: 10.1080/01621459.2018.1562932 (PMC7034331; doi:10.1080/01621459.2018.1562932)
Supplement: Supplemental Material [file UASA_A_1562932_SM9181.zip › UASA_A_1562932_Supplement/voibayes_supp.zip!/voibayes_supp/voibayes_extraplots.pdf]

Value of Information: sensitivity analysis and research design in Bayesian evidence synthesis

Supplementary figures

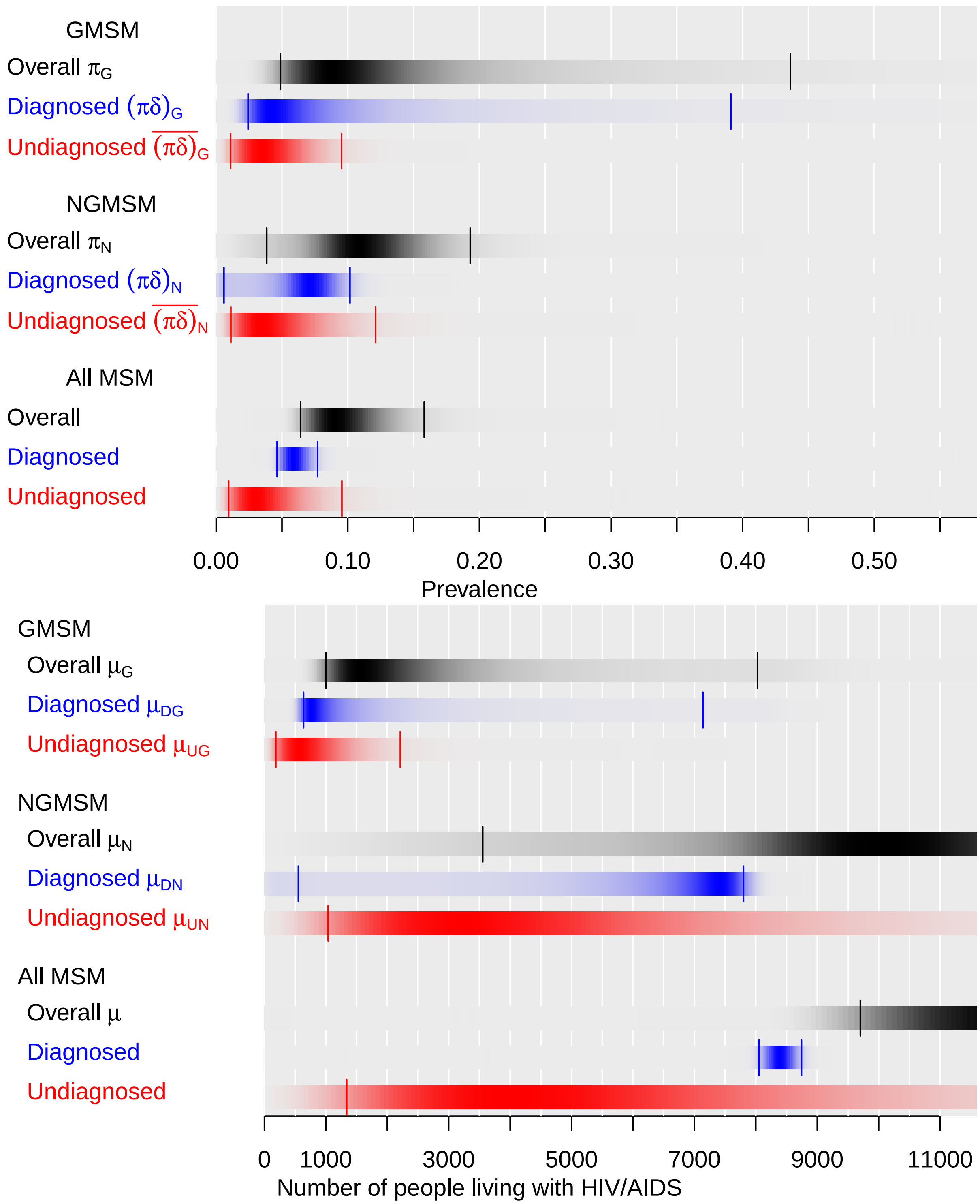

Figure 1: Posterior distributions of HIV prevalence (top) and numbers of MSM living with HIV/AIDS (bottom), London 2012. Darkness within each strip proportional to posterior density, with 95% credible intervals indicated. Alternative assumption (a): undiagnosed prevalence from GUM Anon only

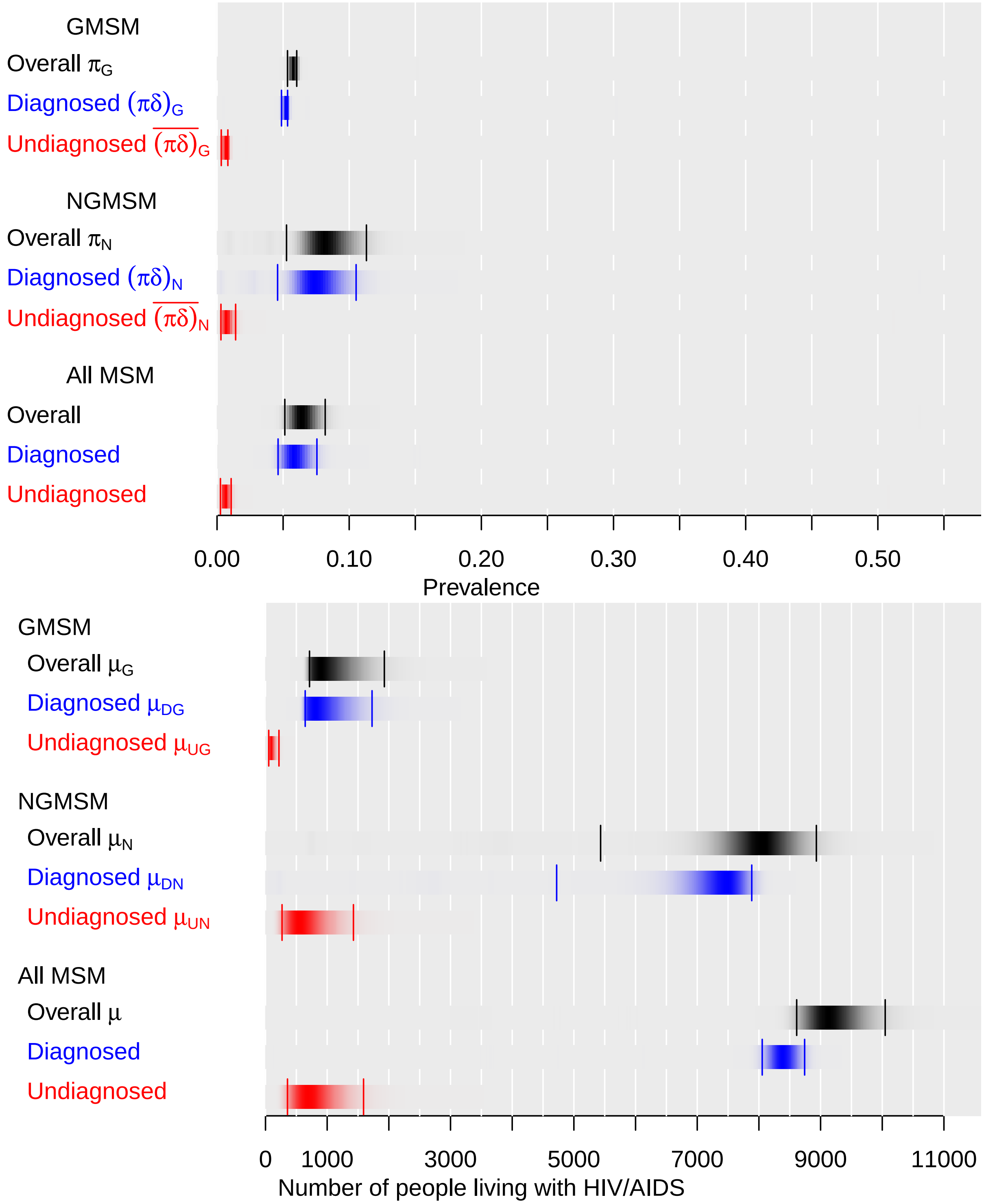

Figure 2: Posterior distributions of HIV prevalence (top) and numbers of MSM living with HIV/AIDS (bottom), London 2012. Darkness within each strip proportional to posterior density, with 95% credible intervals indicated. Alternative assumption (b): GUMCAD also informs diagnosed prevalence

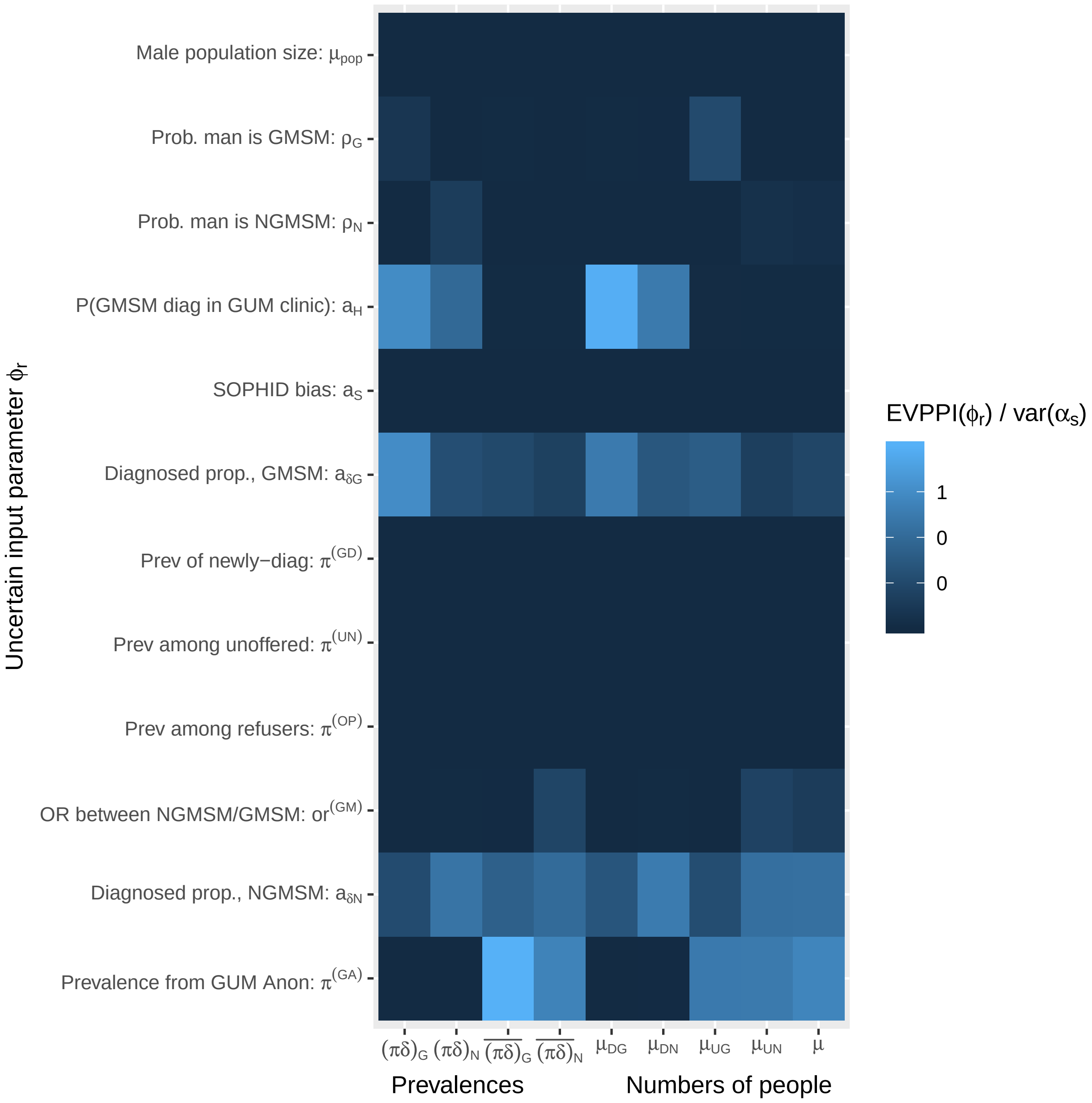

Figure 3: Expected value of partial perfect information in the HIV prevalence model. Alternative assumption (a): undiagnosed prevalence from GUM Anon only

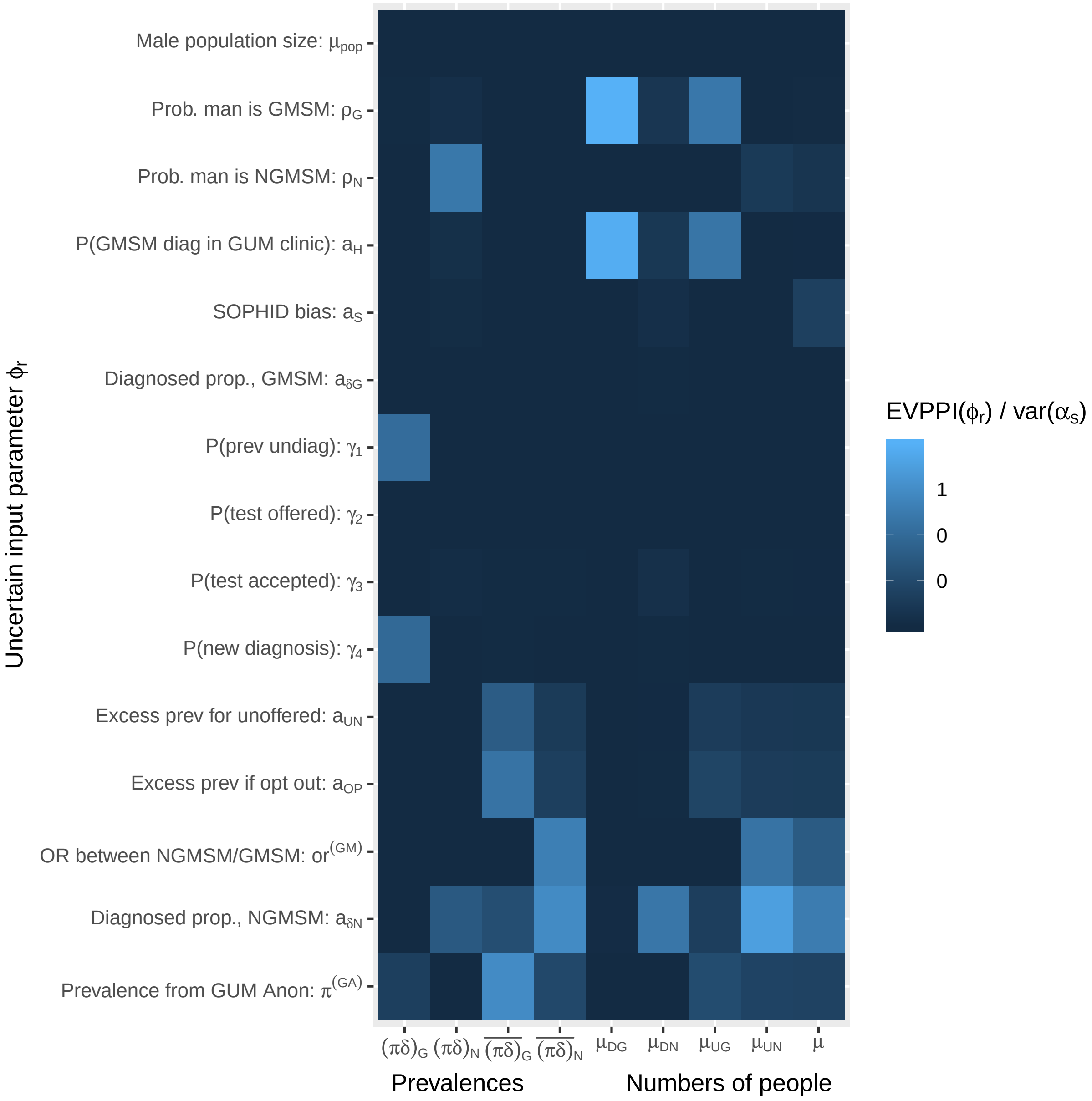

Figure 4: Expected value of partial perfect information in the HIV prevalence model. Alternative assumption (b): GUMCAD also informs diagnosed prevalence
